# Successful Catheter Ablation of Persistent Electrical Storm late Post Myocardial Infarction by Targeting Purkinje Arborization Triggers

**Published:** 2008-11-01

**Authors:** Paul S Thoppil, B Hygriv Rao, S Jaishankar, Calambur Narasimhan

**Affiliations:** Division of Electrophysiology, Department of Cardiology, CARE Hospitals and CARE Foundation, Hyderabad, India

**Keywords:** Electrical storm, Catheter ablation, Purkinje arborization triggers

## Abstract

Drug refractory ventricular tachycardia (VT) occurring as a storm after acute myocardial infarction has grave prognosis. We report a case of a middle-aged lady who presented with drug refractory VT that lead to persistent electrical storm two weeks after an anterior wall myocardial infarction. She underwent a successful catheter ablation of VT followed a few days later by implantation of an AICD. Catheter ablation of the VT could control the persistent electrical storm and the patient was free from a recurrence of VT at three month follow up.

## Introduction

The incidence of incessant drug refractory ventricular tachycardia (VT) after a remote myocardial infarction (MI) is quoted to be 10% [1]. Presentation as an electrical storm occurs in 1%, and is associated with 50% mortality if it manifests within a week after an acute MI [2]. The success rate of catheter ablation of post myocardial infarction VT has steadily increased in recent years on account of improved understanding of the underlying mechanisms that cause ischemic VT. We report a patient who presented in persistent electrical storm two weeks after acute MI and successfully underwent successful catheter ablation of arborizing purkinje triggers near the border-zone of MI where the VT was being initiated.

## Case Report

**Figure 1:**
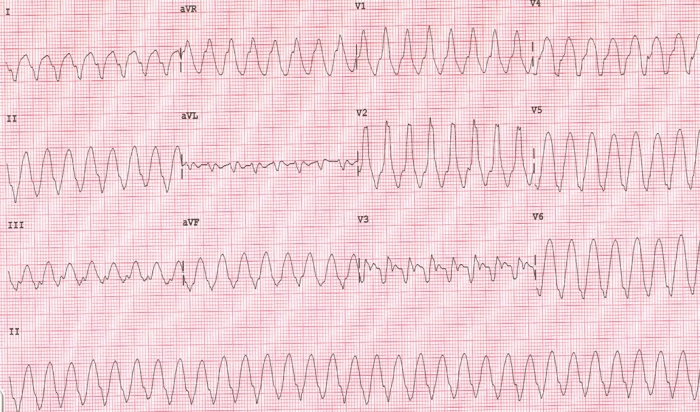
Twelve lead ECG during VT (Monomorphic VT, at a rate of 180/mt, RBBB morphology, Northwest axis).

A 62 years old lady was admitted to an outside facility with evolved anterior wall myocardial infarction and had undergone rescue angioplasty and stenting of single vessel disease in left anterior descending coronary artery (LAD) under cover of Eptifibatide. Ten days after the stenting she developed recurrent episodes of monomorphic VT (at 180/mt, RBBB morphology, North West axis, Figure 1) and presented in an electrical storm unresponsive to multiple antiarrhythmic drugs (IV beta blockers, amiodarone, lidocaine and magnesium), DC shocks, and intraaortic balloon counterpulsation. Overdrive ventricular pacing was performed which was able to restrict VT to around 20 episodes over the previous 24 hr period. The episodes of ventricular tachyarrhythmia were poorly tolerated haemodynamically. An echocardiogram during sinus rhythm had revealed regional wall motion abnormalities in LAD territory and severe LV Dysfunction (LVEF 30%). A check angiogram disclosed a patent stent; there was no thrombus or new lesions. Serum biochemistry, arterial blood gas parameters and thyroid profile were normal. She was referred to our center for feasibility of catheter ablation of VT.

**Figure 2:**
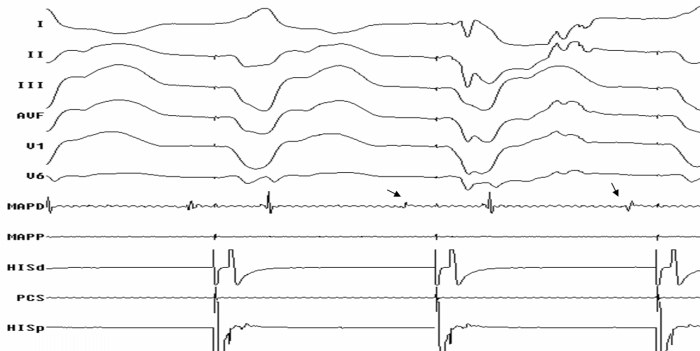
Surface ECG and intracardiac tracings during substrate mapping showing late diastolic potentials (Bold Arrows) in MapD catheter.

**Figure 3:**
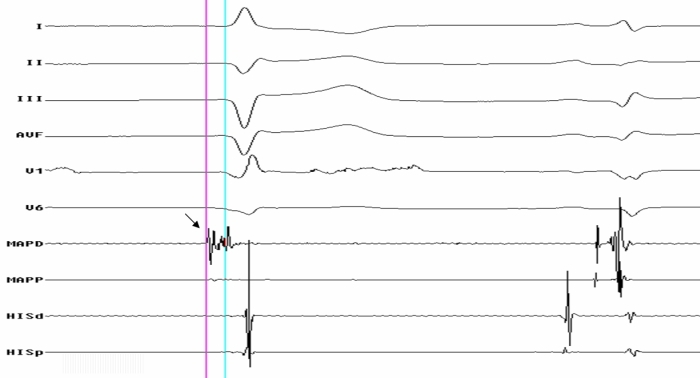
Surface ECG and intracardiac tracings during substrate mapping showing purkinje potential (Bold Arrow) preceding 'V' in first spontaneous ectopic beat, but not detectable in the beat of sinus origin.

**Figure 4:**
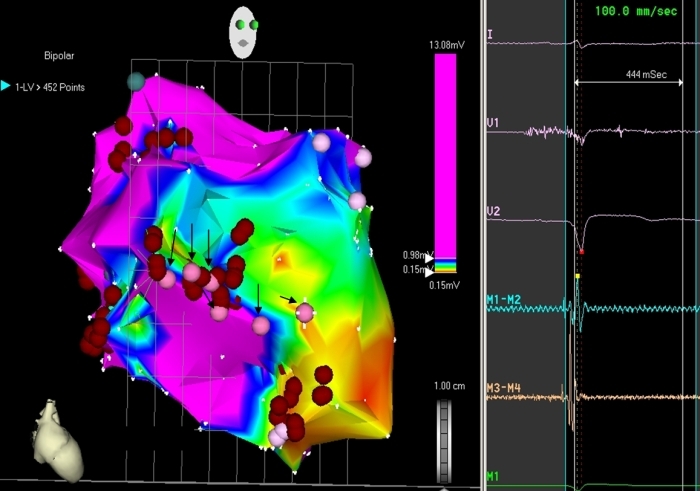
Electroanatomic Bipolar voltage map of LV in RAO projection demonstrating a large scar in anterolateral LV. The voltage map delineates the region of scar border zone. The red tags represent the sites recording late diastolic potentials and pink tags (bold arrows) represents the sites recording discrete Purkinje potentials triggering the VT, which had survived  the Infarct (Successful ablation sites).

She underwent an emergency electrophysiology study during which many attempts at cardioversion and overdrive pacing failed to control the storm. Access to the left ventricle was achieved retrogradely across the aortic valve. LV mapping was performed using a non-fluoroscopic electroanatomic system (CARTO, Biosense Webster Inc, USA) and 7.5F Navistar F curve irrigated tip catheter. A bipolar LV voltage map was created to define the scar and border zone of MI. The infarct area was defined during sinus rhythm by electrograms with an amplitude ≤ 1.5 mV; dense scars were defined by electrograms with an amplitude ≤ 0.5 mV. Once the map was completed, amplitude scale was adjusted (0.2 to 0.5 mV), setting the value for scar at ≤ 0.2 mV to identify conducting channels within the scar area. They were marked out as corridors of continuous electrograms differentiated from the surrounding scar tissue by a higher amplitude, bounded by two scar areas, or by one scar area and the mitral annulus, and connected to normal myocardium by at least two sites [3]. A large scar at anterolateral wall and apex of LV was mapped and the infarct area was completely encircled. RF lesions were delivered using an irrigated tip catheter at 30 W power, 43ºC temperature with an irrigation rate of 20 to 30 mL/min. Whenever a conducting channel was identified along the border zone of scar, a RF lesion was immediately applied to transect the conducting channel. Following the initial ablation of conducting channels and of sites of LV tagged by late diastolic potentials (Figure 2), the  VT storm terminated temporarily but the monomorphic VT could still be reinduced on programmed ventricular stimulation during isoprenaline infusion. Pace mapping at various endocardial LV sites did not produce an ideal match of clinical VT and hence that course was not pursued further. A further substrate mapping of LV revealed *a discrete Purkinje potential preceding spontaneous ventricular ectopic beat* (which was not evident in sinus beats ), suggesting a driver role in the sustenance of the tachycardia (Figure 3). RF lesions were delivered at the LV sites where Purkinje potential preceded the ventricular activation by ≥ 30 ms during ventricular ectopic beats and during tachycardia (Figure 4). After this targeted ablation of Purkinje potential triggers, VT became noninducible with aggressive programmed ventricular stimulation during isoprenaline infusion. A single chamber ICD was implanted a week later and she was discharged in a stable condition. No recurrence of VT or ICD shocks occurred in the two month follow-up.

## Discussion

Electrical storm, defined originally as = 3 distinct episodes of VT/VF within 24-hour period that occurs in ICD recipients, can rarely occur in the few weeks following AMI [4]  and when  becomes persistent, is very difficult to treat. Nademanee et al [2] have pointed out the superiority of sympathetic blockade (left stellate ganglionic blockade and ß-blockade) over antiarrhythmic drug therapy (Lidocaine, Procainamide, Bretylium). The 1 week mortality rate with antiarrhythmic drug therapy was significantly higher than with sympathetic blockade (82% Vs 22%) [2]. In the same study the 1 year VF-free survival for sympathetic blockade group was 67% versus 5% for antiarrhythmic drug therapy group but the VT burden during follow up was not clearly stated. The overall morality rate of patients with electrical storm after AMI remains still high even with antiarrhythmic drug therapy and sympathetic blockade. SMASH-VT trial results revealed that after substrate based catheter ablation there was 65% reduction in delivery of therapy for VT in patients with a history of AMI who received ICDs for the secondary prevention of sudden death [5]. The acute success rate of catheter ablation of recurrent VT after myocardial infarct by combined contact and non contact mapping has been reported to range between 67% and 77% [6].

RF ablation of VT soon after myocardial infarction is more difficult and often challenging, but a need for an attempt at ablation always exists since the mortality rate remains high even with combination antiarrhythmic drug therapy and left stellate ganglionic blockade. Both activation mapping and approaches during sinus rhythm have been shown to be effective methods for ablation of VT. Previous studies conducted on arrhythmias soon after experimental acute myocardial infarction have suggested different kinds of mechanisms responsible for its occurrence. They include enhanced automaticity of subendocardial Purkinje fibers that survived myocardial infarction [7], triggered activity arising from delayed afterdepolarizations [8], and reentry [9]. The damaged border zone area of the scar resulting from MI has been demonstrated to play a crucial role in forming the substrate that sustains macro-reentry and monomorphic VT [10,11]. In such a VT, activation and entrainment mapping allows determination of the critical isthmus of slowed conduction that sustains VT and thus facilitates successful ablation of VT. Activation mapping during the arrhythmia was technically challenging in our patient as she became haemodynamically unstable during sustained VT. Mapping could be performed by ventricular pacing at constant cycle length (550 ms) and later by substrate mapping once VT terminated to sinus rhythm. Ablation after prolonged activation mapping and pace-mapping of endocardial LV was not successful in producing sustained sinus rhythm. It was only after application of RF lesions at the LV sites which demonstrated purkinje potentials preceding ventricular activation that a stable sinus rhythm could be achieved and no VT could be reinduced.  Interestingly, the Purkinje fibers   have been demonstrated as capable of surviving transmural infarction in experimental models, leading to a speculation that their proximity to the endocardium allows imbibition of nutrients from intracavitary blood [7]. The surviving Purkinje fibers that cross the border zone of the MI demonstrate triggered activity, heightened automaticity, and supernormal excitability, which when coupled with prolongation of the action potential duration in this area, may result in the milieu for triggering VT [12,13].

Incessant ventricular tachyarrhythmia soon after MI leading to drug-resistant electrical storm is a rare but technically challenging entity. A search for various triggers that initiate refractory VT should be meticulously made to enhance the success rates of catheter ablation of the VT and to use it as a bailout therapy in these patients.
